# Star-Branched Polyamides as the Matrix in Thermoplastic Composites

**DOI:** 10.3390/polym14050942

**Published:** 2022-02-26

**Authors:** Karina C. Núñez Carrero, Manuel Herrero, María Asensio, Julia Guerrero, Juan Carlos Merino, José María Pastor

**Affiliations:** 1 Foundation for Research and Development in Transport and Energy (CIDAUT), Parque Tecnológico de Boecillo, 47051 Valladolid, Spain; manuel.herrero.villar@alumnos.uva.es (M.H.); marase@cidaut.es (M.A.); julgue@cidaut.es (J.G.); juamer@eii.uva.es (J.C.M.); 2 Department of Condensed Matter Physics, Escuela de Ingenierías Industriales, University of Valladolid, Paseo del Cauce, 59, 47011 Valladolid, Spain; jmpastor@fmc.uva.es

**Keywords:** star-branched polyamide, easy-processing material, thermoplastic composite, ultrasonic welding

## Abstract

The aim of this study is the preparation of star-shaped branched polyamides (sPA6) with low melt viscosity, but also with improved mechanical properties by reactive extrusion. This configuration has been obtained by grafting a tri-functional, three-armed molecule: 5-aminoisophthalic-acid, used as a linking agent (LA). The balance between the fluidity, polarity and mechanical properties of sPA6s is the reason why these materials have been investigated for the impregnation of fabrics in the manufacture of thermoplastic composites. For these impregnation processes, the low viscosity of the melt has allowed the processing parameters (temperature, pressure and time) to be reduced, and its new microstructure has allowed the mechanical properties of virgin thermoplastic resins to be maintained. A significant improvement in the ultrasonic welding processes of the composites was also found when an energy director based on these materials was applied at the interface. In this work, an exhaustive microstructural characterization of the obtained sPAs is presented and related to the final properties of the composites obtained by film stacking.

## 1. Introduction

In recent years, special attention has been given to the research of more sustainable polymeric materials to replace the thermosetting matrix of composites based on fiber fabrics. The main problems associated with the use of thermoset composites are related to their low recyclability, as this matrix cannot flow once the composite is manufactured due to the irreversible cross-linked network that is formed. It is also important to note that these matrices have high toxicity, high cost [1] and very low production rates (long molding and curing times). In addition to the problem of sustainability and cost-effectiveness, thermoset composites have very little ability to integrate with other elements to create more complex and functional components through the traditional welding process [2,3].

The use of thermoplastic matrices in the manufacturing of composites has been recently discussed in the literature to increase process rates [4,5,6,7]. These matrices can melt and solidify in very short times, which translate into very low cycle times and greater economic profitability. Nevertheless, thermoplastic matrices are highly viscous and have very low polarity, which hinders good impregnation of the matrix into the mesh fiber, forming an incompatible system with low adhesion and thus, producing composites with poor mechanical properties. In the past years, our research group has been working on modifying different thermoplastic matrices to find ways to overcome this issue, through the rheological modification of recycled polyester [8,9].

Thermoplastic polyamides (PA) have good mechanical performance, good thermal properties, corrosion resistance, chemical inertness and abrasion performance, which makes them a highly demanded polymer present in a great variety of industrial sectors [10,11]. Over the last two decades, academics and industry have tried to modify the rheological behavior of polyamides depending on their end use. In this sense, there has been an increase in the number of publications developing star conformation polyamides [12,13,14]. These structures are formed by linear chains linked to the central core through the functionalized points. The star-like molecular structures are a spatial conformation of the polymeric chains that allow the exploitation of the occupied volume with a more homogeneous and narrower distribution of molecular weights. This translates into materials with controlled rheology, greater fluidity, greater stickiness and lower melting temperatures compared to a linear polymer of the same molecular weight [15]. Although there are many previous works on the synthesis of star-like structures, in our work we have studied for the first-time star-shaped PAs as an alternative to traditional thermosetting resins in the manufacture of composites.

It has been reported in the literature that different comonomers with different functionalities can be used to obtain star-shaped polyamides by polymerization, and these include: poly-amidoamine dendrimers [16,17], poly(propyleneimine) dendrimers [18,19] and multi-functionalized aromatic compounds [20,21,22]. The effect of different polymerization approaches has also been studied: anionic ring-opening polymerization [23], cationic ring-opening polymerization [13,14] and the traditional hydrolytic polymerization [18,22], although hydrolytic polymerization required long reaction times (>3 h) and high temperatures (>230 °C). On the other hand, anionic and cationic polymerization processes present the advantage of shorter times and lower temperatures, but also the disadvantages of very strict reaction conditions due to the sensitivity to moisture and impurities [15,24].

Regardless of the methodology used, the authors reported sharp changes in thermal and/or rheological behavior with the insertion of multi-functionalized moieties. However, just small changes were reported on the mechanical properties of the star-branched polyamides [15,21]. This occurs as a result of the low comonomer contents that can be added during polymerization processes, where the addition of the multi-functionalized moieties in the medium produces the statistical break between the amine and acid groups of the monomer. In this paper, we discuss a reactive extrusion process to solve this problem. In this way, the polyamide is already polymerized and consequently only the terminal groups are capable of linking with the comonomer (small molecule of 5-aminoisophthalic acid), allowing the addition of higher amounts of liking agent (0.5–5 wt.%). 

Some authors have used reactive extrusion to obtain branched PAs by making use of chain extenders such as anhydride, oxazoline, epoxys, etc. [25,26,27,28,29]. Although these chain extenders can improve mechanical properties, they significantly increase the viscosity of the polymer. On the other hand, there are studies where reactive extrusion is used for the synthesis of caprolactam by anionic ring-opening polymerization, with the objective of generating long-chain branches to increase the molar mass. This can be achieved by using a linking agent that can connect two chains in a linear matter, first to double its molar mass and then later generate 3- or 4-armed polymers in a controllable manner [30]. The significant viscosity increases induced by these changes mean that none of these matrices can be used for impregnation applications; therefore, a short-branched star polyamide must be assessed.

Therefore, the aim of this study is the preparation of star-branched polyamides by reactive extrusion to be used as a matrix in the manufacture of composites with fiber fabrics, finding a balance between the processability and functionality of the final composite. This work analyzes the relationship between the microstructure of the sPA6 matrix studied and the final mechanical properties of the obtained composite by the film stacking method, as well as the capacity of this new thermoplastic matrix to improve the welding procedures of these materials by ultrasonic methods.

## 2. Materials and Methods

### 2.1. Material

The 5-aminoisophthalic acid (98% purity) used as a linking agent (LA) was supplied by Cymit Quimica, (Barcelona, Spain) and used without further purification. The commercial polyamide used in the study was a low-viscosity polyamide (Akulon K222-D) from DSM (Heerlen, The Netherlands). Biaxial glass fabric of 520 g/m^2^ by SAERTEX (Saerbeck, Germany) was used in the fabrication of the composites by the film stacking process.

### 2.2. Reactive Extrusion Process

Akulon K222-D (PA6) and 5-aminoisophthalic acid (LA) were dried under vacuum at 80 and 60 °C, respectively, for 16 hours before processing. Once the materials were dried, four star-branched polyamides (sPA6) with 0.5, 1, 2.5 and 5 wt.% of LA (0.5 sPA6, 1 sPA6, 2.5 sPA6 and 5 sPA6, respectively) were obtained using a co-rotating twin-screw extruder model, Leistritz 27 GL. The extrusion temperature profile ranged from 250 to 260 °C and the material was extruded at 30 rpm (about 3 minutes of residence time). In addition to mixing and transport elements, shear and flow reverse elements were introduced to improve the PA6–LA interaction and increase reaction times. LA was fed through a side port of the extruder, to ensure that it was in contact with the molten PA6. The neat PA6 was submitted to the same extrusion process to ensure the same thermal history. All the materials were pelletized and dried prior to the injection process. 

### 2.3. Injection Molding Process of sPA6

The pelletized samples were injected using a Krauss Maffei KM 200 injection molding machine. The temperature of the cylinders was 250 °C and the mold temperature was 60 °C. The injected specimens were type 1A (according to ISO 527-1) for the tensile tests. These specimens were later cut following the standard requirements for all the rest of the specific characterizations (Charpy impact and heat-distortion temperature tests). Additionally, a spiral mold was used to study the flow of the obtained sPA6.

### 2.4. Characterization of sPA6

#### 2.4.1. Morphological Characterization

^1^H NMR spectra were collected with an Avance400 NMR spectrometer (Brucker, Germany) using H_2_SO_4_ + 10% D2O as a solvent. Prior to the X-ray diffraction (XRD) experiments, the samples were heated and maintained at 230 °C for 5 min to remove the thermal history of these samples. Then, the samples were quenched in liquid nitrogen and subsequently maintained at room temperature for 24 h. XRD analysis of the original and sPA6 was performed using a D8ADVANCE wide-angle X-ray diffractometer (Brucker, Germany) at room temperature. XRD data were collected from 10 to 30° of 2θ. Molecular weight distributions of obtained samples were determined by GPC using HPLC-PDA. GPC columns (PLgel 10 μm MIXED-B) were used with hexafuoroisopropanol (HFIP) as an eluent at 25 °C. Column calibration was performed using PMMA standards.

#### 2.4.2. Thermal Characterization

The melt and crystallization temperatures, as well as the heats of fusion and crystallization of the star-branched polyamides, were measured by differential scanning calorimetry (DSC), with a Mettler Toledo DSC 851e (Columbus, OH, USA) in the temperature range from 25 to 300 °C at a heating rate of 10 °C min^−1^ under nitrogen flow. The selected enthalpy of fusion for perfect PA6 crystal was 190 J/g [17]. Thermogravimetric analysis (TGA) was used to determine the decomposition temperature of the obtained sPA. Thermograms were obtained in nitrogen atmosphere at a heating rate of 10 °C min^−1^ using a Mettler-Toledo TGA851 (Columbus, OH, USA).

#### 2.4.3. Rheological Characterization

The melt flow index (MFI) was determined by measuring the polymer fluidity in the molten state at 230 °C and 2.16 kg according to ISO 1133. For solution viscosimetry, polyamide samples were dissolved in units of g/dL (six different concentrations of 0.2–1 g/dL) in formic acid (90%). Then, they were placed in an Ubbelohde viscometer and efflux times were measured. The same procedure was used for formic acid used as a solvent. The intrinsic viscosities’ values were calculated according to ASTM D 2857. From these values, the viscosity molecular weight (*M_v_*) of the polyamides was calculated using the Mark–Houwink–Sakurada equation:

(1) $$\left[ƞ\right]=KM_{v}^{a}$$

In this equation, “*K*” and “*a*” values are specific for fixed conditions of polymer type, solvent and temperature. For the conditions mentioned above (formic acid and 25 °C), 0.463·10^−4^ dL/g and 0.97 were used as values of “*K*” and “*a*”, respectively. Finally, capillary rheometry experiments were carried out with a CEAST Twin Bore 5000 equipped with a 30/1 (mm/mm) round die capillary at melt temperatures of 230 and 260 °C. Time sweep tests were conducted at a 1 Hz frequency over 1000 s. The shear viscosity was obtained in the shear rate range of 100−100,000 s^−1^. Dynamic oscillatory tests were performed in the linear viscoelastic region, with an angular frequency ranging from 0.1 to 628 rad/s.

#### 2.4.4. Mechanical Characterization 

Tensile test: The Young´s modulus and the elongation at break were measured at room temperature with an Instron model 5500R60025 (Barcelona, Spain) at a speed of 1 mm min^−1^ and 50 mm/min^−1^, respectively, according to ISO 527-1. For each material, seven specimens were tested, and mean values of the mechanical parameters were calculated. Charpy impact test: the notched Charpy test values were measured in a Resil 6957 impact pendulum at room temperature according to ISO 179. Heat-distortion temperature was measured in a CEAST HDT-3VICAT P/N 6911/000, using a 1.8 MPa load, according to ISO 75. For each material, three specimens were tested, and mean values were calculated. In order to obtain specific mechanical properties, the density of sPSA6 was determined by means of liquid immersion under the standard ISO 1183-1.

### 2.5. Obtaining of the Thermoplastic Composite Based on sPA

#### 2.5.1. Calendering Process

In order to obtain 2 mm-thick consolidated sheets and considering the weights of the fabrics to be used, it was necessary to manufacture films with an average thickness of 160 µm. For this purpose, the calendering parameters were optimized. A calendering process of sPA6 and 2.5sPA6 was performed using COLLIN equipment, Model Techline CR72T, (Barcelona, Spain) and the processing conditions used were: (a) rolls’ speed: 10–30 rpm, (b) rolls’ temperature: 10 °C, (c) extrusion temperature: 240–245 °C and (d) screw speed: 120–125 rpm. Samples were collected during the steady-state operation. 

#### 2.5.2. Compression Molding (Film Stacking)

Successive stacking of thermoplastic films and glass fiber textile was performed. The laminates were manufactured using six sheets of sPA6 film and five layers of glass fiber textile. Once stacked, a Teflon-coated film was placed on the top and bottom faces, sealed between two metal sheets and placed in the press (240 °C for polyamides) for two minutes. At this stage, it is necessary to ensure that both sheets are in contact with the press plates, but no pressure is necessary. As a result of this pre-treatment, the “preforms” could be cut on a band saw to the size of the mold, without defects in the lamination sequence.

Once the “preform” was cut, it was introduced into the mold, with the exact size and bordered with rubber bands to avoid displacements and contain plastic leaks. Finally, the mold was introduced into the press, where different compaction times and temperatures were used to evaluate the influence of these factors. In all cases, a value of 12 bar of pressure was exerted. Once the proposed time was reached, refrigeration was introduced and demolding took place when the thermocouples registered 30 °C. Consolidated thermoplastic composite sheets of 200 × 200 mm were obtained.

#### 2.5.3. Thermoplastic Composites’ Characterization

The tensile properties and Charpy impact absorption were measured following the same standards and test conditions as for the mechanical characterization of thermoplastic matrices. For the characterization, specimens were obtained from the consolidated thermoplastic composite sheets by machining.

#### 2.5.4. Welding Tests of Composites Obtained with Matrix sPA6

The welding equipment used was a 40 kHz LPX ultrasonic generator, with a power of 500 W. All the tests were carried out in energy mode. The validity of the bonding of the welded composites was studied with tensile tests. Energy directors fabricated with sPA6 by additive manufacturing (Fused Filament Fabrication) were used between the composites in order to concentrate the energy supplied by the welding equipment at the welding interface. Prior to additive manufacturing, sPA6 filaments were manufactured using a FILABOX EX6 (Barre, VT, USA), extruder at 245 °C.

## 3. Results and Discussion

### 3.1. Obtaining and Characterization of Star-Branched PA

**Figure 1:**
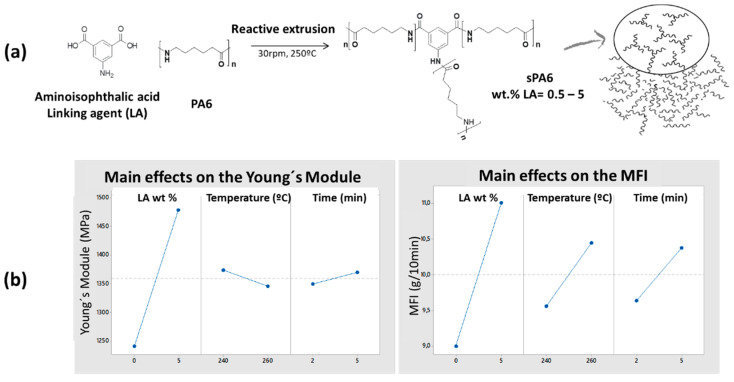
(**a**) Representation of the synthesis process of 3-arm star-type polyamides. (**b**) Choosing the main experimental parameters conditioning the fluidity and mechanical properties of sPA6 by DoE.

The design of the reactive extrusion process for the preparation of sPA was based on the condensation of the polyamide end groups with the multifunctional groups of the aromatic compound (aminoisophthalic acid or LA). It was hypothesized that the insertion of the aromatic structure into the polyamide chains will afford the final polymer a higher stiffness (better mechanical properties) but will also break the linear distribution of the polymer chains, decreasing the melt viscosity and crystallinity (see Figure 1a). 

In order to choose the experimental extrusion parameters, a first design of experiments (DoE; Taguchi design and data processing with Minitab17 statistical software) was carried out (Figure 1b). A linear relationship between the increase or decrease in Young´s modulus or MFI (fluidity) and parameters such as temperature, amount of agent and residence times was found. In order to find a balance of sPA6 properties, the following parameters were chosen: temperature profile ranging from 250 to 260 °C at 30 rpm, and LA < 5 wt.%.

#### 3.1.1. Morphological Characterization 

***Nuclear magnetic resonance (NMR)***

**Figure 2:**
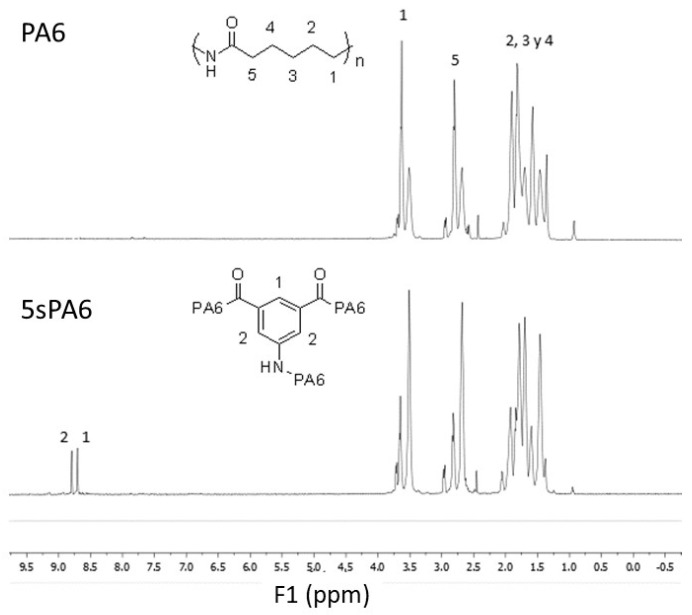
^1^H spectra of the raw PA6 and the star-branched polyamide 5sPA6.

In Figure 2, the NMR spectra of 5sPA6 and its virgin analogue are shown. Due to the impossibility of dissolving the polyamide in common solvents, it was necessary to fragment it using deuterated water. The structure cannot be clearly discerned either due to the high acidity of the deuterated solvent. However, it is possible to observe the signals due to the 5-aminophthalic acid in the range between 8.5 and 9 ppm [15]. This demonstrates the insertion of the modifier, it is also noteworthy that each -CH_2_- of the polymer chain shows different shifts, with lower-intensity signals appearing. This phenomenon may be due to the additivation carried out by the manufacturers during the synthesis of the commercial polymer used as a starting point in this study. Besides, the grafting of a highly polar molecule onto the hydrocarbon chains of the polyamide and furthermore creating cross-linking points and branching affects the chemical environment of the original CH_2_ bonds of the polyamides. The new interactions are responsible for both the shift and the change in the intensity of the NMR signals.

***Wide-Angle X-ray Scattering (WAXD)***

**Figure 3:**
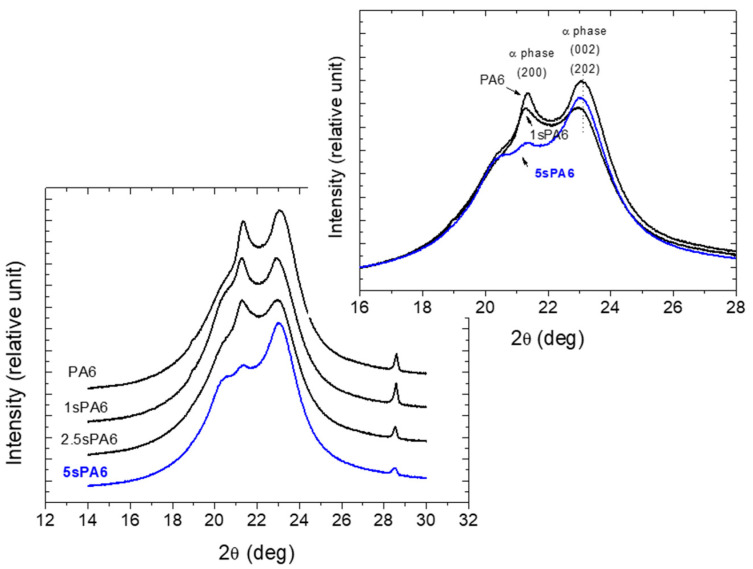
WAXS curves of the star-branched polyamides.

In order to investigate the crystalline morphologies of sPA6 samples, WAXS studies were carried out. In Figure 3, the sPA6 samples showed the characteristic peaks associated with the α phase at 2θ = 20.98° and 23.02° that were related to the monoclinic structure [31]. The intensity of the diffraction curve of the sPA6 samples decreased with respect to virgin PA6, it was most markedly in the case with higher amounts of LA. This suggests that the crystallinity degree and the size of the crystal of sPA6 samples decreased as the number of branch points increased [23,32]. It is worth noting that the reflections at 2θ ≈ 23° and 24° in PA6 refer to the H-bonded planes [33]. The decrease in this intensity also suggests that the polyamide stars have changed the interaction between them and their arrangement in the crystalline system. 

***Size Exclusion Chromatography (SEC)***

**Figure 4:**
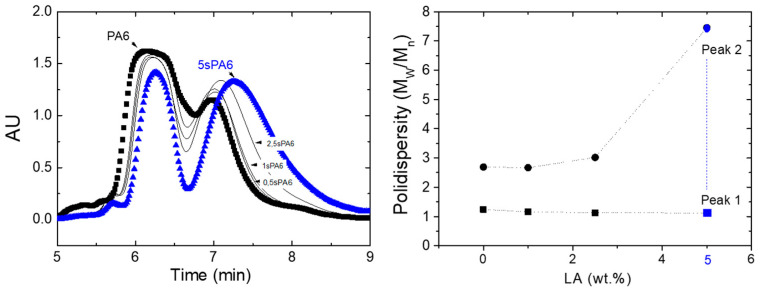
(**Left**) SEC curves of the star-branched polyamides. (**Right**) Polydispersity of the peaks for the bimodal distribution of the star-branched polyamides.

The thermal and mechanical properties, as well as the processing behavior of the polymers, depend on their molecular weight and its distributions. In order to study these properties, SEC analyses were performed, and curves are shown in Figure 4. It can be observed how the increase of LA produced two different phenomena: On the one hand, the peaks were displaced at higher retention times, which means lower number and weight average molar mass (Mn and Mw, respectively, see Table 1). On the other hand, the detected signal decreased for the first peak (highest times) and increased for the second (lower times), see Figure 4 (left). Both phenomena are translated into a drop in the molecular weight of the samples as the LA content increases.

Regarding the dispersity, it is considered as an indicator of the distribution of individual molecular masses in a complex sample. From data of Mn and Mw, collected from the software, the polydispersity (Mw/Mn) for each peak was calculated and the results are presented in Figure 4 (right). According to this interpretation, the increase of the linked agent produced an increase in the dispersity of peak 2, which corresponded with the lower molecular weight fraction, but no major changes were observed for the first peak. This phenomenon can also be observed in Figure 4 (left), where the increase of the linked agent broadened the distribution of the second peak (bigger interval for the retention time), but the width of first peak was preserved. Finally, star conformations produced by reactive extrusion have lower molecular weights and, although slightly less polydisperse overall (see Table 1), have a wide distribution of molecular weights in the predominant low molecular weight zone, when compared to virgin PA (see Figure 4). This is a consequence of the fact that the grafted 5-aminoisophthalic acid has not acted as a chain extender but as an insertion point for short branches.

#### 3.1.2. Thermal Properties

**Figure 5:**
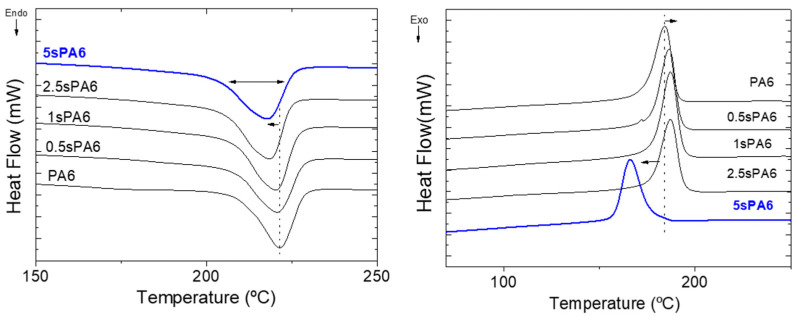
DSC thermograms of the star-branched polyamides’ (**left**) melting and (**right**) crystallization curves.

Once it was demonstrated that the star conformation obtained by reactive extrusion has an important influence on the molecular weight and on the crystallization of PA6, thermal properties associated with the main transitions of the polymer were studied. Table 1 contains the thermal properties of the samples determined from heating and cooling thermograms (see Figure 5). The first important observation of the thermograms was similarity between all the samples in terms of peak morphology and position. However, a slight but a continuous decrease of the crystallinity, with the increase of LA, was observed. As discussed, the obtained star conformation resulted in less regular chains, and the LA unit in the PA6 matrix decreased the intensive interaction of hydrogen bonds between neighboring molecules; for these reasons, the ability of the polymer to crystallize is gradually reduced [23,34]. These results are in accordance with the conclusions of the WAXS and SEC analyses.

Low concentrations of LA in the sPA6 structure allow the crystallization temperature to increase slightly (~2 degrees), meaning that these lower molecular weight and less crystalline structures have the ability to crystallize earlier than virgin PA6, possibly due to their higher molecular mobility. However, this slight tendency is reversed when the amounts of LA are higher than 2.5%, then a sharp drop in T_c_ is observed. It may be attributed to the branch points that cannot be inserted into the crystal lattice [24]. 

The decrease of the T_m_ and T_c_, with the increase of the linking agent, has been reported before in some papers about star-branched structures [13,14,15,23]. In these studies, the drop in these temperatures was sharper even with lower contents of LA. The steeper decrease may be a consequence of the process of obtaining them (in situ polymerization), where high amounts of comonomer means shorter branched chains [24,35]. Conversely, in this study, only terminal groups of the already polymerized polyamide are able to link with the LA due to the use of an extrusion process. In this case, the length of the chains was not reduced as much as in the polymerization process, and consequently the decrease in T_m_ was controlled. The controlled decreased of T_m_, T_c_ and crystallinity as well as their broader peak would offer potential advantages in material processing and elucidate the existence of branched structures, which could disturb the original arrangement and hydrogen bonding interactions of the polymer chains [16,23,32].

It is important to note that TGA measurements showed that these structural changes are also associated with the loss of thermal stability of sPA6 with respect to virgin PA. In this sense, high LA contents decreased the maximum decomposition temperature by more than 10 °C. This should be considered for the final application of these materials.

Finally, in order to quantify the amount of LA that did not react during the reactive extrusion and that could be present in the sPA6 as a filler, conditioning its thermal properties, it is important to mention that when the TGA tests were performed, none of the sPA6 curves showed decomposition at temperatures below 315 °C, which corresponds to the decomposition temperature of pure acid (LA). On the contrary, all of them started to decompose above 440 °C, typical decomposition temperatures for PA6 chains, so we can state that the residual portion of LA is very small and practically undetectable. This result was also confirmed in the SEC tests, where no low molecular weight population was detected.

Furthermore, the changes in thermal and rheological properties are so significant that we can be sure that there was a very high rate of LA grafting into the structure.

#### 3.1.3. Rheological Characterization 

***Melt flow index (MFI) and intrinsic viscosity***

Although the loss of molecular weight in star conformations has been proven, it is important to study its implication for the processability of these new materials. In this sense, the viscosity of the star-branched polyamides was studied with various techniques, and the obtained values are shown in Table 1. The MFI is the most widely used procedure for the routine characterization of the resistance to flow on melted polymers. However, MFI results are not a well-defined rheological property and some authors advise against their use for polyamides and polyesters due to their ability for water absorption [36]. To avoid these problems, the traditional way to measure the viscosity of polyamides is the viscosimetry technique (by solution). Table 1 shows the results of the fluidity obtained by both techniques (MFI and Viscosimetry).

**Figure 6:**
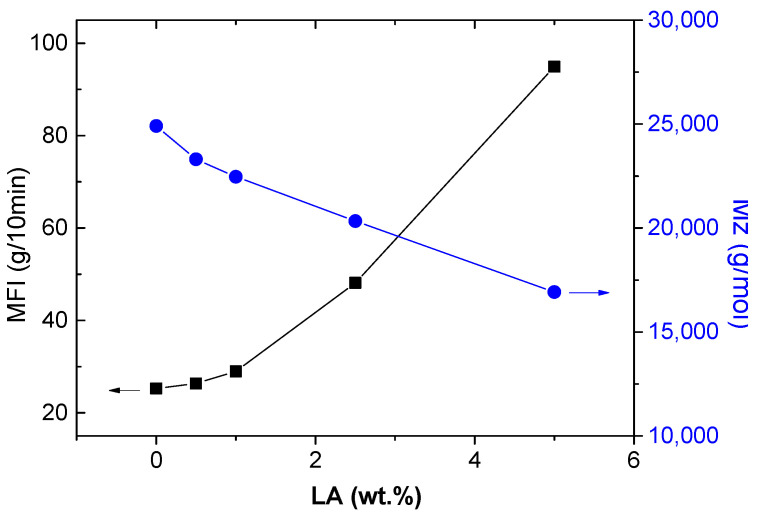
MFI values and viscosity molecular weight (M_z_) of the star-branched polymers versus the content of 5-aminoisophthalic acid.

The rise of the trifunctional agent (LA) within the polymer matrix produced a quick increase in the MFI values. These results mean a higher mass of polymer flowing during the test and consequently lower melt viscosity. The same conclusion was observed for the intrinsic viscosity, where high amounts of LA produced a decrease of the test time. This means a decrease in solution viscosity and consequently a lower-viscosity molecular weight. From Figure 6, it can be seen that the branching effect was stronger on melt viscosity than solution viscosity. The special configuration of this type of structure obtained by reactive extrusion allows high fluidity to be achieved without a significant loss of molecular weight. This is a great advantage compared to star polyamides obtained by polymerization. This molecular weight maintenance will have an impact on the mechanical behavior of the materials, as shown below.

***Capillary rheology***

**Figure 7:**
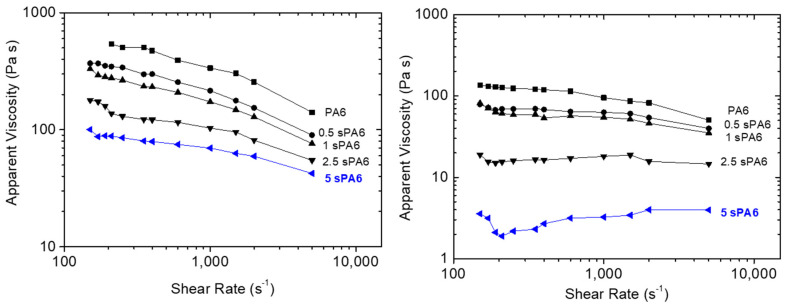
Apparent viscosity of the samples by capillary rheometry versus shear rate: (**left**) 240 °C and (**right**) 260 °C.

In order to know the rheological behavior of the star-branched polymers at different shear rates (typical for extrusion and injection molding processes), capillary rheometry was carried out, and the curves are presented in Figure 7. Regardless of the test temperature, the strong effect of branching on the final viscosity of the materials can be observed. The addition of the LA caused a significant viscosity drop associated with an apparent loss of shear rate dependence at higher temperatures (260 °C). This phenomenon has already been reported by several authors [14,37].

It is known that the shear behavior in the melt of star-shaped macromolecules differs from that of their linear analogues, and that it essentially depends on the number and length of the arms [14,37]. It was reported that long-chain branching (LCB) had a significant effect on the rheological properties of polymers, and even a small amount of LCB could lead to a great increase in extensional viscosity, shear viscosity and elasticity resulting from the enhancement of molecular entanglement, and consequently, a higher draw ratio could be achieved during the subsequent hot stretching [34]. The sPA6 obtained by reactive extrusion shows the opposite behavior, a significant loss of viscosity with shear rate, which is due to the grafting of short-chain branching (SCB) with a controlled loss of overall molecular weight that does not generate significant molecular entanglement. This was confirmed by the data provided by the SEC tests.

**Figure 8:**
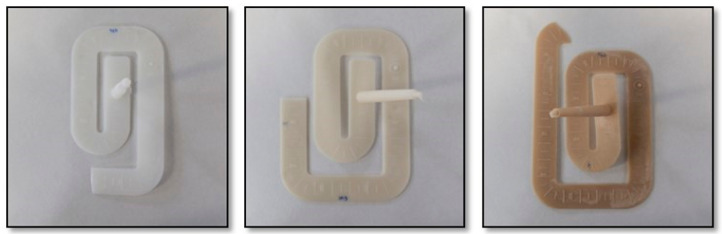
Spiral specimens for PA6 (**left**) and star polyamides modified with 2.5% (**center**) and 5% (**right**) 5-aminophthalic acid.

This reduction in melt viscosity with the applied shear stress is an advantage from an industrial point of view, where lower viscosity means softer parameters during the processing of star-branched polyamides to final applications, as long as the final mechanical properties are maintained. In order to verify this rheological advantage of the sPA6, these materials were injected into a spiral mold at fixed pressure. In Figure 8, it can be seen how the increase in the amount of LA produced a greater advance of the melt inside the mold before reaching the limit pressure, demonstrating the lower viscosity of the star-type polyamides. Additionally, the color change of these materials as the amount of LA increased can be observed.

**Figure 9:**
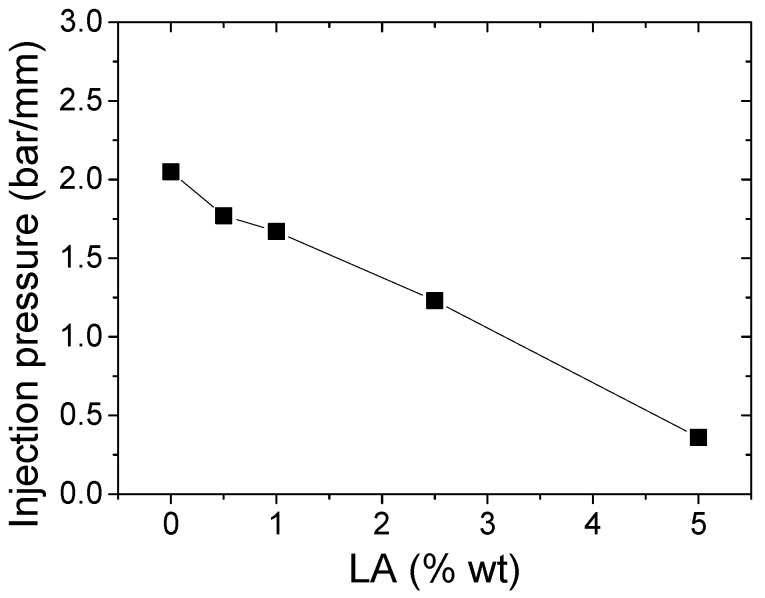
Pressures required to advance 1 mm in the spiral mold of sPA6 with different amounts of LA.

To demonstrate the benefits of this behavior, Figure 9 shows the pressure required to advance the material inside the mold versus the LA content. This graph shows that as the final concentration of the modifier increased, the required pressure decreased, linearly.

#### 3.1.4. Mechanical Properties

**Figure 10:**
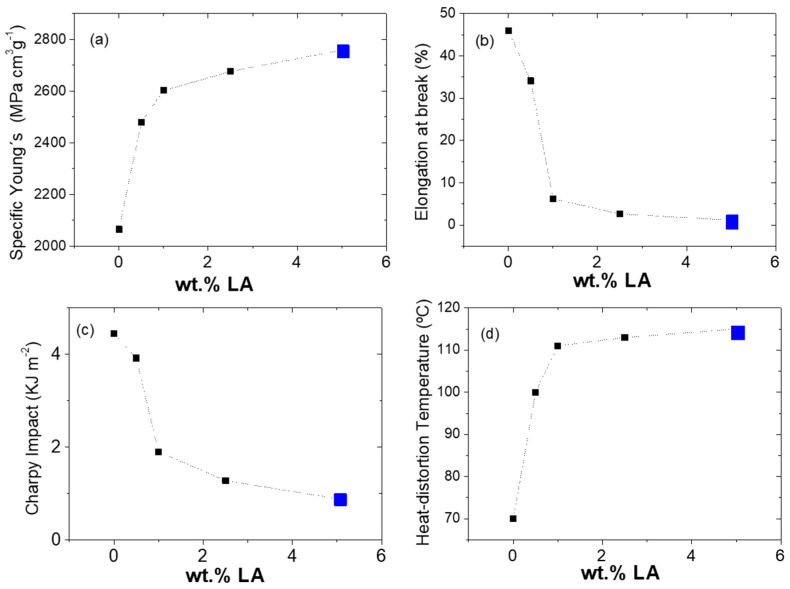
Specific mechanical properties of modified polyamides (sPA6) under different preparation approaches as a function of the 5-aminoisophthalic acid content: (**a**) Young’s modulus, (**b**) elongation at break, (**c**) Charpy impact test and (**d**) heat-distortion temperature.

It is not only important to demonstrate the rheological improvements achieved with this configuration, but also that there was no significant loss of mechanical properties. A detailed list of the mechanical properties of the star-branched polyamides is provided in Table 2 and an overview of specific properties in Figure 10. The first important observation is the correlation of the specific Young’s modulus with the amount of LA (Figure 10a). The modulus showed a sharp increase even with a low amount of LA. The star-branched polyamides with 0.5 and 5 wt.% of LA showed increases of 30% and 45% with respect to the neat polyamide. 

These results are due to the more compact nature of the branched polymers and the inability of star-shaped molecules to undergo as much reorientation and molecular “slippage,” thus agreeing with previously published literature [18]. It is important to note that the grafted SCBs are defects of the crystalline system, so it is not the crystallinity that is responsible for the rigidity obtained, but the existence of aromatic groups in the molecular chain. Therefore, sPA6 needs more force to break its chemical bonds and overcome its intermolecular forces than linear PA6. It was shown that the increase in stiffness is proportional to the amount of LA added, however a plateau (%LA > 2.5) where there is practically no change was observed. For this reason, in this work LA was studied at low concentrations.

In contrast, Figure 10b shows the observed negative correlation between the amount of LA and the elongation at break. The star-branched polyamides showed a sharp decrease of the deformation, and only the samples loaded with 0.5 and 1 wt.% of LA presented some deformation before breaking. The same trend was observed during the impact test (Figure 10c), where the insertion of the linking agent produced a reduction of the energy capable of being absorbed by the materials. Possibly, the low breaking strength and impact energy absorption of sPA6s are mainly due to their low molecular weight and relatively short branched chains.

The results of the mechanical properties tests of the studied materials indicate that the viscosity of PA6 can be significantly reduced while maintaining or improving its mechanical performance through star branching with the use of an appropriate concentration of 5-aminoisophthalic acid.

HDT is the temperature at which the specified deformation is reached by applying a certain load on the polymer and heating it at a certain rate. It is one of the key physical parameters to characterize the heat resistance of polymers [37]. Figure 10d shows the striking increase of HDT values with the increase of LA within the polymer structure. The highest HDT of 115 °C was found for the polymer with 5 wt.% of LA, which corresponds to an increase of 72% compared to neat PA6. This is indeed a remarkable result. The increase in HDT in PAs is generally attributed to the use of reinforcing fillers that induce the stiffness of the system (nanoparticles, short fibers, polymer blends, etc.) [38], including the amount of moisture [39], which in the case of sPA6 can be affected by the increase in polarity. However, this result is mainly attributed to the significant stiffness in star structures with low LA concentrations due to the strong hydrogen bonds between the matrix and the aromatic group surface [40].

Some authors claim that the increase in HDT may be due to the formation of more perfect crystals [41]. From the results obtained from the microstructural characterization, we can state that the LA induces the transformation of α phase crystals, increasing Young’s modulus values.

### 3.2. Characterization of Thermoplastic Composites Based on Star-Branched PA

**Figure 11:**
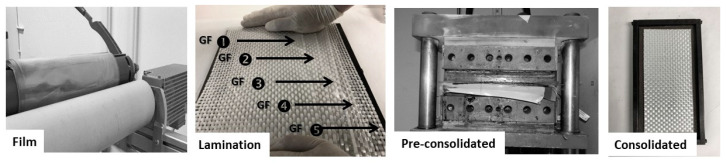
Images of the forming process: film manufacturing of 2.5sPA6, lamination sequence, pre-consolidation and final specimen after compaction.

In order to evaluate the suitability of the matrix-fiber systems, PA6 or 2.5sPA6 and glass fiber laminates were prepared, as shown in Figure 11. The 2.5sPA6 was chosen as it had the best balance of mechanical properties and flowability. The consolidation of these composites was carried out using different temperatures (240 and 260 °C) and different times (2, 5, 10, 15 and 30 minutes) in order to evaluate the advantages of the star-like structure in improving processing conditions. These laminates were mechanically characterized by Charpy impact and tensile tests.

**Figure 12:**
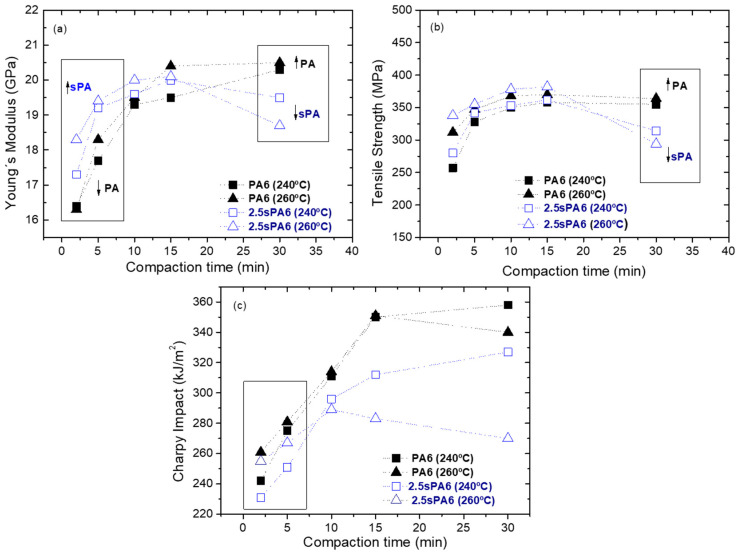
Results obtained from the characterization of composites of PA6 and 2.5sPA6 by film stacking at 240 and 260 °C: (**a**)Young’s modulus, (**b**) tensile stress and (**c**) Charpy impact test.

Regardless of the temperature, at low consolidation times, the composite manufactured with star-branched PA had a higher stiffness in comparison to virgin PA6, but this tendency was reversed at high consolidation times (see Figure 12a). This demonstrates the significant improvement of the interaction between the matrix and the fiber with the new microstructure obtained. This results in a significant effective stress transmission at the interface which offers high tensile strength [42]. Additionally, the higher fluidity of sPA6 allows penetrating the mesh textile more easily in less time. However, it can be seen that consolidation times longer than 15 minutes led to a decrease in the values. This is associated with the degradation that the polymer undergoes at high consolidation times during the forming stage. This phenomenon is more appreciable in the modified materials (sPA6), which coincides with the results of the thermal tests carried out. 

At low consolidation times, the stiffness of composites made with sPA6 as a matrix was higher than those manufactured with virgin material, at 260 °C. However, it can also be observed that at times between 10 and 15 minutes, there was practically no difference in the Young’s modulus and the maximum stress of the composite obtained with 2.5sPA6 at 240 and 260 °C. Therefore, a lower temperature (240 °C) can be used for longer consolidation times (see Figure 12a,b).

In the case of the impact tests (see Figure 12c), the observed behavior was completely different. For this property, the best values were obtained for the structural composites obtained with raw PA6, regardless of temperature and consolidation times. This is a consequence of their higher toughness and higher resistance to thermal degradation. 

**Figure 13:**
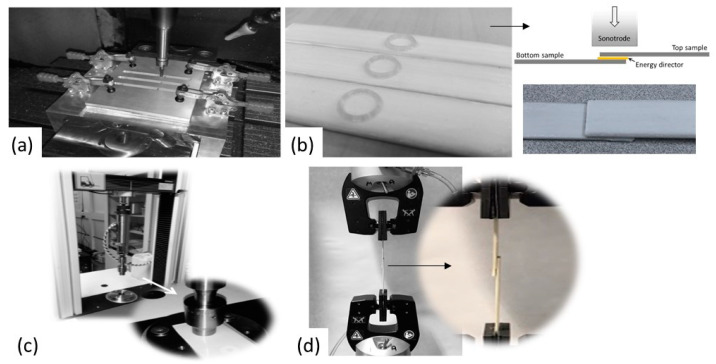
Welding process. (**a**) Milling of 2.5sPA6 and raw PA6 composite specimens, (**b**) deposition of 2.5sPA6 as an energy director on the specimens by FFF, (**c**) ultrasonic welding with cylindrical sonotrode and (**d**) mechanical test to evaluate weld strength.

As a consequence of the balance of the obtained properties, a thermoplastic composite with 2.5sPA6 as the matrix was fabricated at a low temperature (240 °C) and low consolidation times (10 min) by the film staking process. It was mechanized to carry out ultrasonic welding tests. Its corresponding composite with the unmodified PA6 matrix, at the same processing conditions, was also fabricated in order to make comparisons and evaluate improvements. Figure 13 describes the procedure carried out.

During the welding process, a sonotrode applies high-frequency (40 kHz), low-amplitude vibrations to the weld interface, while simultaneously applying a constant static pressure. The interface generates heat through surface friction and viscoelastic heating. An energy director, based on 2.5sPA6 deposited by FFF, is placed at the interface to focus the heat generation [43]. The energy directors are shaped so that they undergo high oscillating strains and, as a result, they heat up preferentially [44]. In this research, a circular shape was chosen.

The test results are shown in Table 3. Regardless of the use of the energy director, the specimens of the composite made with the star-branched polyamide showed a stronger weld (more than 30% compared to virgin PA6). This is due to the fact that this modified polyamide is more polar and less crystalline, as demonstrated in microstructural tests. This significantly improves the efficiency of locally re-melting the thermoplastic to weld individual parts together [43]. In addition, in these low consolidation times (10 min), the composite made with 2.5 sPA6 achieved better impregnation on the glass fabric due to its high fluidity, i.e., a larger area of the polymer was in contact with the fiber and with the welding energy. It should be noted that in any thermoplastic composite welding process, lack of intimate contact or wetting at the welding interface would preclude the formation of the welded joint [45]. Undoubtedly, the rheological properties achieved in the star-branched polyamides play a significant role in this regard.

Related to the use of the energy director, the weld strength of the unmodified polyamide-based composites improved markedly when a star-branched polyamide was applied as the energy director at the interface (by almost 20%). This demonstrates the potential of these materials to be used as welding energy concentrators due to the increase in interfacial friction of the contacting surfaces [46] and also their high compatibility with pure PA.

The weld strength was further improved when both the composite matrix and energy director were fabricated with 2.5sPA6. In recent years, some authors have attributed these improvements to the viscoelastic response of the material during the welding process [47,48]. In this sense, the obtained microstructures appear to have a viscoelastic heating and dissipation response, under cycle loading, superior to a linear PA6.

## 4. Conclusions

This study was focused on the design of a special grade of polyamide (star shape) by reactive extrusion for application in fabrics’ impregnation. Specifically, the polyamides from this study were used for obtaining structural thermoplastics by film stacking and to be welded by ultrasonic processes. RMN, DSC and WAXS analyses provided indirect evidence to confirm the existence of a star-branched structure and a significant transformation of the crystalline system compared to the virgin PA6. These changes had a major influence on the rheological, thermal and mechanical response of these materials. 

The results have shown that it is possible to manufacture a thermoplastic composite with a sPA6 matrix, with higher weld capability by ultrasonic processes, while maintaining the mechanical properties of a composite made with conventional PA6. Additionally, this composite has easy processing (lower processing times and lower temperatures), as the high fluidity of these materials allows it.

Finally, this material can be easily obtained by reactive extrusion from a commercial polymer with the addition of very small quantities of a linking agent. The aromatic character of this graft played a decisive role in increasing the stiffness, the polarity and the insertion of short branches into the structure. This has led to the balance of the required rheological and mechanical properties.

## Figures and Tables

**Table 1:** Molecular weights, thermal and rheological properties of the star-branched polyamides.

Sample	Mw ^a^ (g/mol)	Mn ^a^ (g/mol)	Mw/Mn	X_c_ ^b^ ± 1 %	T_c_ ^c^ ± 0.5 °C	T_m_ ^d^ ± 0.5 °C	T_d_ ^e^ ± 0.5 °C	MFI ^f^ (g/10min)	[ƞ] ^g^ (dL/g)	M_z_ ^g^
PA6	90,778	72,981	1.24	37.6	191.7	221.0	460.2	25	0.85	24,902
0.5sPA6	85,405	74,890	1.14	36.3	193.3	219.3	459.6	26	0.80	23,304
1sPA6	84,617	74,197	1.14	35.0	193.9	219.0	457.1	29	0.77	22,462
2.5sPA6	81,109	71,549	1.13	34.4	192.6	218.0	450.7	48	0.70	20,330
5sPA6	77,120	68,335	1.13	31.0	176.0	217.0	448.6	95	0.59	16,921

^a^ Measured by SEC (Mw: weight average molecular weight, Mn: number average molecular weight and Mw/Mn: overall polydispersity). ^b^ X_c_: Crystallization rate, ^c^ T_c_: crystallization temperature, ^d^ T_m_: melt temperature (%X_c_, T_m_ and T_c_ by DSC), ^e^ T_d_: decomposition temperature by TGA, ^f^ MFI: melt flow index, ^g^ [ƞ]: intrinsic viscosity and M_z_: viscosity molecular weight obtained from intrinsic viscosity using the Mark–Houwink–Sakurada equation.

**Table 2:** Mechanical properties of raw polyamide and star-branched polyamides.

Sample	Young’s Modulus (MPa)	Elongation at Break (%)	Charpy Impact (kJ/m^2^)	HDT (°C)
PA6	2084 ± 21	46 ± 14	4.5 ± 1	70 ± 1
0.5sPA6	2815 ± 64	34 ± 9	3.9 ± 1	98 ± 2
1sPA6	2956 ± 44	10 ± 3	1.9 ± 0.5	109 ± 1
2.5sPA6	3061 ± 68	<1	1.3 ± 0.5	113 ± 1
5sPA6	3160 ± 88	<1	0.9 ± 0.3	115 ± 1

**Table 3:** Weld strength of composites manufactured with raw PA6 and 2.5sPA6. Evaluation of the improvements due to the energy director made with 2.5sPA6.

Specimens		Welded Test Specimens		
Thermoplastic Composite	Energy Director Based on 2.5sPA6	Max. Strength	Displacement	Tensile Strength
		(N)	(mm)	(MPa)
2.5sPA6 matrix	No	2059	0.88	12.2
2.5sPA6 matrix	Yes	2134	0.91	14.5
Raw PA6 matrix	No	1782	1.32	8.4
Raw PA6 matrix	Yes	1898	1.74	10.1

## Data Availability

The data presented in this study are available upon request from the corresponding author.
